# Cardiomyogenesis Modeling Using Pluripotent Stem Cells: The Role of Microenvironmental Signaling

**DOI:** 10.3389/fcell.2019.00164

**Published:** 2019-08-09

**Authors:** Amanda Leitolis, Anny W. Robert, Isabela T. Pereira, Alejandro Correa, Marco A. Stimamiglio

**Affiliations:** Stem Cell Basic Biology Laboratory, Carlos Chagas Institute, FIOCRUZ-PR, Curitiba, Brazil

**Keywords:** cardiomyocytes, pluripotent stem cell, secretome, cell differentiation, maturation

## Abstract

Pluripotent stem cells (PSC) can be used as a model to study cardiomyogenic differentiation. *In vitro* modeling can reproduce cardiac development through modulation of some key signaling pathways. Therefore, many studies make use of this strategy to better understand cardiomyogenesis complexity and to determine possible ways to modulate cell fate. However, challenges remain regarding efficiency of differentiation protocols, cardiomyocyte (CM) maturation and therapeutic applications. Considering that the extracellular milieu is crucial for cellular behavior control, cardiac niche studies, such as those identifying secreted molecules from adult or neonatal tissues, allow the identification of extracellular factors that may contribute to CM differentiation and maturation. This review will focus on cardiomyogenesis modeling using PSC and the elements involved in cardiac microenvironmental signaling (the secretome – extracellular vesicles, extracellular matrix and soluble factors) that may contribute to CM specification and maturation.

## Introduction

Pluripotent stem cells (PSC), both embryonic stem cells (ESC) and induced pluripotent stem cells (iPSC), show strong potential to proliferate and differentiate. PSC have already been successfully differentiated into a number of cell types, including cardiomyocytes (CM) (Murry and Keller, 2008). Key events that regulate lineage commitment can be reproduced *in vitro* and used as a model to study cardiomyogenesis, to generate CMs and produce clinically relevant cell populations, and to evaluate cardiac toxicity or model congenital abnormalities (Kehat et al., 2001; Xu et al., 2002; Laflamme et al., 2007; Kattman et al., 2011; Burridge et al., 2012). Despite the advances in this field, new challenges are emerging, mainly related to cardiac differentiation efficiency and the functional maturation of human PSC-derived cardiomyocytes (hPSC-CM).

This review discusses cardiac differentiation and hPSC-CM maturation approaches that use extracellular components of the cardiac microenvironment. Initially, an overview of hPSC cardiomyogenic differentiation protocols was described, indicating some of the essential signaling pathways that control CM commitment. However, the main focus is to explore the cardiac niche, its components and the strategies developed to mimic its complexity *in vitro*. After a brief description of important signals and interactions available in a tissue niche, we emphasize aspects related to cardiac extracellular matrix (ECM; composition or structure), soluble factors and extracellular vesicles (EVs) that could influence *in vitro* CM differentiation and maturation.

## Overview of Heart Development

The heart is a complex muscular organ composed of several cell types, including CM, smooth muscle cells (SMC), endothelial cells (EC), cardiac fibroblasts (cFB), and cardiac progenitor cells (CPC). Although CM occupy most of the heart volume, they comprise only ∼40% of the total cells. The other 60% largely comprises EC and cFB, however, the percentage of each of them is still not certain (Banerjee et al., 2007; Bergmann et al., 2015; Pinto et al., 2016).

**FIGURE 1:**
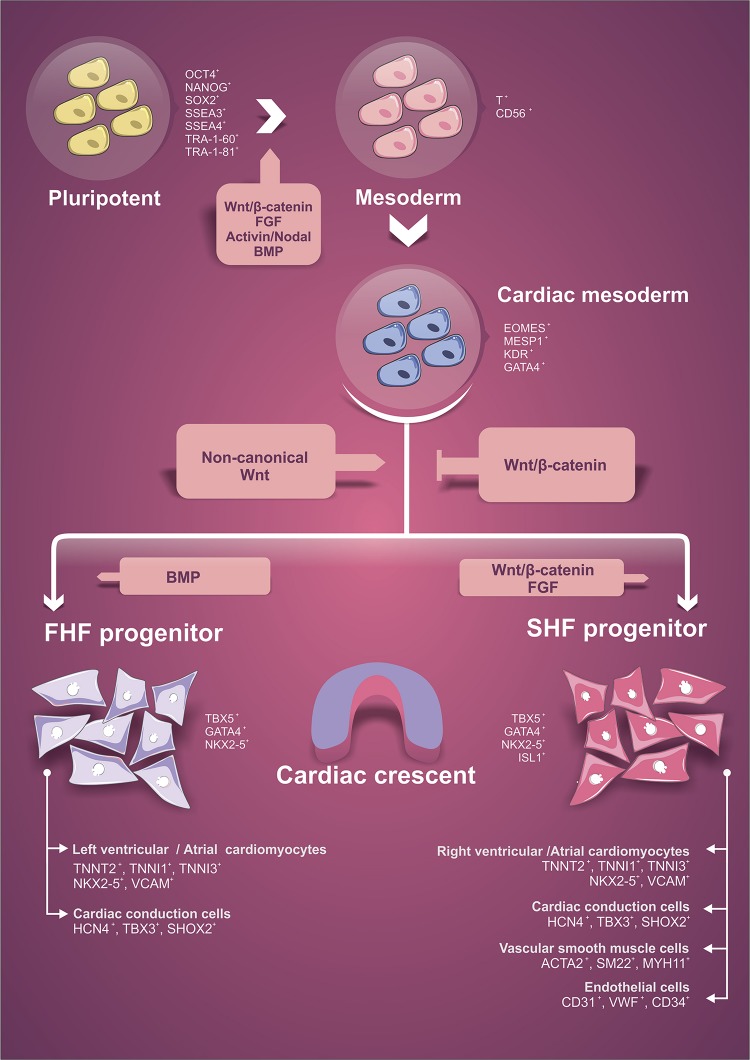
Schematic representation of the initial steps of cardiac lineage commitment. Indication of signaling pathways that influence each differentiation stage and the specific cellular markers expressed during lineage differentiation. FHF, first heart field. SHF, second heart field.

The heart is the first organ to become functional in the vertebrate embryo (Brand, 2003). Although the heart develops early, cardiogenesis is a highly regulated process involving differentiation and cellular specialization, spatial integration and coordination of several signaling pathways. Cardiac tissue is mostly derived from the mesodermal layer and the induction to the cardiomyogenic phenotype depends on signals derived from adjacent layers, such as endodermal and ectodermal cells (Wagner and Siddiqui, 2007; Sun and Kontaridis, 2018). The signaling factors modulated over heart development include members of bone morphogenetic proteins (BMPs), Activin and NODAL, fibroblast growth factor (FGF), and Wingless (Wnt) families (Brand, 2003; Wagner and Siddiqui, 2007; Liu and Foley, 2011; Brade et al., 2013; Sun and Kontaridis, 2018). In Figure 1, we briefly highlight some aspects of embryonic cardiac commitment that will be important to understand and support the *in vitro* differentiation protocols using PSC. The signaling pathways influencing the stages of cell differentiation and the cell markers expressed in these different stages are indicated (Figure 1). For more details about the morphogenesis, signaling pathways and factors involved in this process, see Wagner and Siddiqui (2007), Brade et al. (2013), Sylva et al. (2014), Paige et al. (2015), Sun and Kontaridis (2018).

## *In vitro* Differentiation of hPSC

Cardiac cell fate specification occurs through progressive steps that we are currently able to reproduce at the laboratory. There are three major strategies to derive CM from hPSC: (1) inductive coculture, (2) embryoid bodies, and (3) monolayer cultures. Table 1 summarizes these strategies and their main references [complete reviews can be found in Burridge et al. (2012), Mummery et al. (2012), Denning et al. (2016)].

**TABLE 1:** Three major *in vitro* cardiac differentiation protocols.

**Cardiac differentiation protocols**		
**Strategy**	**Induction method**	**Main References**
**Inductive coculture with END-2** *visceral endoderm-like cells*	direct coculture or END-2 conditioned medium	Mummery et al., 2003, 2007
**Embryoid Body (EB)**	spontaneous differentiation	Itskovitz-Eldor et al., 2000; Kehat et al., 2001; Zhang et al., 2009
	growth factors in defined media (GFs)	Yang et al., 2008; Kattman et al., 2011; Ren et al., 2011
**Monolayer**	growth factors in defined media (GFs)	Laflamme et al., 2007
	small molecules in defined media (SM)	Lian et al., 2012; Burridge et al., 2014

*Their main references are indicated.*

The inductive coculture protocol uses visceral endoderm-like cells (END-2), which play an important role in signaling the adjacent mesoderm in developing embryos, to induce the differentiation of cardiogenic precursor cells. This protocol was developed by Mummery et al. (2003) and improved by them in Mummery et al. (2007). The convenience of the coculture is that it requires few cells and is less time-consuming. On the other hand, the efficiency of the protocol is very low, and it is not commonly used.

Three-dimensional aggregates, known as embryoid bodies (EBs), represent the first structure in which CM could be produced *in vitro*. Using a protocol to derive spontaneous differentiation, contracting structures containing CM and other germ layer derivatives were generated (Itskovitz-Eldor et al., 2000). These EBs could also be transferred to attachment culture plates and give rise to beating areas (Kehat et al., 2001; Zhang et al., 2009). Studies in animal models have helped with valuable information for the optimization of *in vitro* hPSC differentiation protocols (Marvin et al., 2001; Ueno et al., 2007). Cardiomyocyte derivation from hPSC can be manipulated and directed to cardiac lineage by specific elements, such as growth factors known to be involved in *in vivo* heart development (Vidarsson et al., 2010). The same signaling pathways mentioned above as essential for heart development are also used to modulate hPSC differentiation *in vitro*, such as BMP, Nodal, FGF, and Wnt (Filipczyka et al., 2007). Concentration and time of addition or removal of specific factors, such as BMP4, activin A and Wnt modulators, are critical for cardiac lineage specification and were adapted in the protocols to improve efficiency (Yang et al., 2008; Kattman et al., 2011; Ren et al., 2011). A similar idea was applied to the monolayer-based protocol, in which the use of growth factors and other molecules could improve efficiency and would not be interfered by the diffusional barrier present in EBs (Mummery et al., 2012). Using a defined serum-free medium supplemented with BMP4 and activin A, Laflamme et al. (2007) reported better efficiency in CM differentiation in the monolayer system compared to the EB method. Other adaptations were made (Lian et al., 2012; Burridge et al., 2014), including the use of more specific and low cost signaling small molecules, such as CHIR99021 (an inhibitor of glycogen synthase kinase 3), leading to the monolayer protocol becoming the most popular and routinely employed cardiogenic differentiation method (Denning et al., 2016). In addition, the ECM influence was also tested to improve differentiation efficiency. Combining ECM with growth factor signaling in a protocol that uses a double Matrigel layer, so-called matrix sandwich, CM could be generated with high purity (Zhang J. et al., 2012).

Advances in methodologies to direct cardiac differentiation helped to improve efficiency in the protocols, increasing the final percentage of CM (usually cardiac troponin T positive – cTnT^+^). However, the purity of these populations is still a limitation in the field. Some approaches were developed to enrich CM, rather than other cell types. For example, genetic selection strategies were based on the expression of a drug resistance gene or reporter protein gene under the control of a cardiac-specific promoter (Xu et al., 2008; Kita-Matsuo et al., 2009; Elliott et al., 2011). Reporter protein genes could also be applied in flow cytometry sorting, as well as selection by markers from distinct stages of differentiation (Yang et al., 2008; Lin et al., 2012; Den Hartogh et al., 2015). Purification of CM using a Percoll gradient (Xu et al., 2002) and energy metabolism differences (Tohyama et al., 2013) were also established. In addition to the purity of final populations, another limitation of *in vitro* cardiogenic differentiation is that the currently available methods generate a heterogeneous CM population that includes a mix of subtypes, such as ventricular, atrial, pacemaker, and non-contractile cells (Kolanowski et al., 2017; Friedman et al., 2018). Strategies to derive specific cardiac cell subtypes are being developed and could help the demand for therapeutic applications of these cells (Zhang et al., 2011; Karakikes et al., 2014; Devalla et al., 2015; Protze et al., 2016; Lee J.H. et al., 2017).

Another challenge in the field of hPSC cardiac differentiation is related to the maturity of hPSC-CM: most of the protocols generate immature CM. In recent years, a great number of studies have focused on investigating strategies to improve the maturation of hPSC-CM and make them more similar to adult CM (Sartiani et al., 2007; Lundy et al., 2013; Robertson et al., 2013), which are multinucleated (25–30%) with highly organized sarcomeres (I, A, and Z bands, M lines and intercalated disks), T-tubules, high expression of sarcomeric and ion channel genes, fatty acid β-oxidation metabolism, higher contractile force and upstroke and conduction velocities (Yang et al., 2014; Dunn and Palecek, 2018; Machiraju and Greenway, 2019). Among the approaches to achieve this purpose are the prolonged time for culture (e.g., 120–360 days), addition of hormones, metabolites or other soluble factors, mechanical or electrical stimulus, microtissue development, coculture with other cell types (such as SMC, cFB, and EC), stiffness regulation, and tridimensional (3D) cultures with biomaterials or ECM components (reviewed by Yang et al., 2014; Besser et al., 2018; Dunn and Palecek, 2018; Machiraju and Greenway, 2019).

Hence, cardiac differentiation efficiency and, consequently, the purity of the cell population and the immature phenotype of hPSC-CM are among the challenges regarding the use of these cells for drug discovery models, development studies, tissue engineering or cellular therapies. Considering the importance of the microenvironment for cellular behavior, we believe that knowledge about the cardiac niche and the use of strategies to mimic its specificities could improve (or create new) hPSC cardiac differentiation and maturation approaches. Since the coordination of all cellular processes including how the cells participate in the microenvironment is governed, inter alia, by gene expression, transcriptomic and proteomic analyses can contribute to the elucidation of cardiac niche specificities and also help to overcome the challenges of using hPSC-CM.

High-throughput studies have allowed the investigation of the global changes in gene expression at the transcriptional, post-transcriptional and protein levels, contributing with considerable insights about gene regulatory programs that are crucial to control cardiac tissue formation (Table 2). Distinct gene expression patterns drive PSC into specific cell types, consequently, the investigation of sequential stages during differentiation might help to discover essential molecules that determines the final destination of a cell. For instance, Paige et al. (2012) and, more recently, Liu et al. (2017) showed a temporal alteration in chromatin structure when analyzing distinct time-points of *in vitro* cardiomyogenesis. Changes in DNA methylation and post-transcriptional regulation were also described during cardiac differentiation and potentially participate in the modulation of gene regulatory programs (Tompkins et al., 2016; Fu et al., 2018; Pereira et al., 2019). Key regulators of cardiovascular development identified in those studies could be used to improve differentiation protocols, e.g., activating or inhibiting a specific signaling pathway. In addition, the identification of new cell surface proteins, such as the studies of den Hartogh et al. (2016) and Van Hoof et al. (2010), could provide new markers for development stages, cell lineage, cell subtype or maturation. Proteomic approaches also have highlighted important aspects regarding the cardiac commitment process, such as the metabolic and mitochondrial maturation described by Poon et al. (2015), the identification of metabolic and cytoskeletal proteins by Konze et al. (2017b) and the identification of proteins related to specific metabolic process, e.g., ketogenesis, described by Kim et al. (2019). Genes related to the ECM were also shown as modulated throughout the cardiac commitment process (den Hartogh et al., 2016; Pereira et al., 2019), and it will be discussed later in this review.

**TABLE 2:** Summary of transcriptomic and proteomic studies based on hPSC cardiac differentiation.

**Differentiation method**	**Time-points**	**High throughput method**	**Highlights**	**References**
Monolayer in END-2 coculture	Days 0, 1, 3, 6, 9, and 12	Agilent Microarray	Identification and validation of time-dependent gene expression patterns	Beqqali et al., 2006
EB spontaneous differentiation	hESC, CM, and hF heart	Agilent Microarray	hESC-CM promoting recovery from cardiac ischemia reperfusion injury	Cao et al., 2008
EB in END-2 conditioned medium	hESC, EBs, CM-Day 21, fetal heart and adult heart	Illumina microarray	Evaluation of the biological relevance of uncharacterized genes	Xu et al., 2009
EB (GFs)	Days 0, 2, 5, 9, and 14	ChIP-seq and Affymetrix array	Temporal alterations in chromatin structure identify key regulators of cardiovascular development	Paige et al., 2012
EB (GFs) KDRlow/CD166 +	hESC or iPS, M-Day 6, Day 20: CM, SMC, EC	Illumina RNA-seq	Lineage-enriched genes and lncRNAs, RNA splicing isoforms	Li et al., 2015
Monolayer (SM/C)	hESC, D3, D4 and CM-D31	Illumina RNA-seq and MDB-seq	TFs, miRNAs, lncRNAs and methylome	Tompkins et al., 2016
Monolayer (GFs, SM/C) * Day 3 MESP1 + sorting	Days 0, 3, 5, 7, 10, and 14	Illumina Microarray	Regulation of ECM components and new cell surface markers	den Hartogh et al., 2016
Monolayer cardiomyocyte differentiation kit (Thermo Fisher Scientific)	Days 0, 2, 4, and 30	RNA-seq and ATAC-seq	Mapping open chromatin patterns	Liu et al., 2017
Monolayer (GFs)	Days 0, 12 and 20	Illumina Microarray and ChIP-seq	Genetic and epigenetic changes and a role for NR2F2	Pursani et al., 2017
Monolayer (SM/C)	Days 0, 2, 5, 15, and 30	Illumina single-cell RNA-seq	Cardiomyocyte hypertrophy and maturation	Friedman et al., 2018
Monolayer (SM/C)	Days 0, 5, 14, and 45	Single-cell RNA-seq	Single-cell heterogeneity	Churko et al., 2018
EB (SM/C)	Days 0, 2, 5, 15 and 30	Illumina RNA-seq and RRB-seq	TFs, lincRNAs and DNA methylation changes	Fu et al., 2018
EB (GFs)	Days 0, 1, 4, 9, and 15	Illumina RNA-seq polysome	Polysomal RNAs and post-transcriptional regulation	Pereira et al., 2018
Monolayer (SM/C)	Days 0, 2, 5, and 14	RNA-seq and ATAC-seq	Interplay of local and global chromatin structure on gene regulation	Bertero et al., 2019
Monolayer in END-2 coculture	differentiated hESC, enriched populations of hESC-derived CM and primary hF CM	SILAC-based quantitative MS	Identification of cell surface proteins for antibody-based selection	Van Hoof et al., 2010
EB (GFs)	hESC, hESC-VCMs, hF-VCMs, and hA-VCMs.	2D-Differential-In-Gel Electrophoresis followed by MS	Metabolic and mitochondrial maturation	Poon et al., 2015
Monolayer (GFs, SM/C)	Days 0, 5, and 14	label-free quantitative	Identification of known and unknown regulatory proteins	Hofsteen et al., 2016
Monolayer (SM/C)	Days 0, 20, and 35	SILAC-labeled	Metabolic and cytoskeletal proteins	Konze et al., 2017b
Monolayer (SM/C)	Days 0, 7, and 15	SILAC upon PAL-based capture of sialylated glycoproteins (glyco-proteomic)	Global proteomic, sialo-glycoproteomic, and glycomic characterization	Konze et al., 2017a
Monolayer (SM/C)	Days 0, 5, and 15	Three-plex tandem mass tag labeling	Identification of proteins associated with branched chain amino acid degradation and ketogenesis	Kim et al., 2019

*CM, cardiomyocytes; END-2, visceral endoderm-like cells; EB, embryoid body; EC, endothelial cells; GFs, growth factors; hF, human fetal; hA, human adult; iPSC, induced pluripotent cell; lncRNA, long non-coding RNA; M, multipotential cardiovascular progenitor cells; miRNA, microRNA; MS, mass spectrometry SMC, smooth muscle cells; SM/C, small molecules and chemical compounds; TFs, transcriptional factors; VCM, ventricular cardiomyocytes.*

## Tissue Microenvironment: Composition, Interactions, and Importance

Stem cells are modulated by microenvironmental cues in which they are embedded. This surrounding microenvironment is called the stem cell niche and is able to support cell maintenance and regulate the expansion or differentiation of stem cell populations. Thus, the stem cell niche was defined as an anatomical structure that includes cellular and acellular components, which integrates local and systemic factors that regulate stem cell behavior (Jones and Wagers, 2008). Since first being described by Schofield (1978), the niche concept has been expanded; it currently includes many components: the stem cell itself and tissue stromal cells; the ECM and related molecules; the secreted factors and extracellular vesicles (EVs); the blood vessels that carry systemic signals and cells (as immune regulators); the neural stimuli; oxygen concentration; and physical cues, such as shear stress (Jones and Wagers, 2008; Ferraro et al., 2010).

**FIGURE 2:**
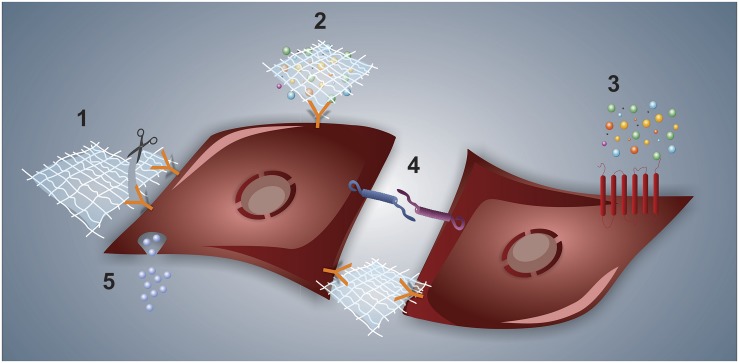
Types of interactions at the cell–niche interface. In (1), a representative interaction between the cell and ECM is shown, whereas the matrikine-receptor interaction is pictured alongside the scissor, which represents matrix proteases. The ECM can also bind to and present a given trophic factor to the cell, as depicted in (2). The typical cytokine–receptor interaction is shown in (3). Cell-to-cell interactions are illustrated in (4), both by interacting membrane adhesive proteins and through ECM-integrin-mediated interaction. Finally, extracellular vesicle-driven signaling is represented in (5).

Cell signaling in the niche should be pictured as a result of multiple interactions and stimuli involving cell-to-cell connections, adhesive and de-adhesive ECM proteins, soluble trophic factors, and EVs. Figure 2 depicts some of the interactions that might occur along with niche networking. The complex netting of fibrillar proteins, proteoglycans and glycoproteins that compose the ECM acts in support and structure cells in the niches. In addition, ECM proteins and bioactive fragments released from the ECM by enzymatic degradation (the so-called matrikines) trigger cellular responses through interaction with cell receptors (Rozario and DeSimone, 2010; Ricard-Blum and Salza, 2014). Additionally, the ECM provides signals to the cells through its mechanical features (e.g., stiffness and elasticity) and its ability to function as a reservoir of growth factors and other bioactive elements. These secreted soluble factors may act locally or may diffuse throughout the niche, generating a concentration gradient (Jones and Wagers, 2008; Brizzi et al., 2012) that activates/inhibits signal pathways in the cells through autocrine/paracrine routes. Furthermore, another important player to consider on the niche site is the EVs that load different types of cargo (e.g., proteins, small RNAs, lipids) and function in intercellular communication (Stik et al., 2017; Durand et al., 2018).

Distinct stem cell populations are arranged in unique and specific tissue microenvironments (Xin et al., 2016), within which the way stem cells behave depends on the interactions between specific cells and niche elements. Regarding the heart, cardiogenic niches are highly dynamic, presenting different functionalities and characteristics according to the stage of development of the heart and its physiological state. During the early stages of development, the elements of the cardiogenic niche play roles related primarily to cell expansion and specification, controlling the size and shape of cardiac structures. Subsequently, the niche elements stimulate the completion of differentiation and maturation of cardiac cells (revised by Christalla et al., 2012). In the adult heart, however, the current understanding of the cardiac niches is limited. Fatih Kocabas et al. (2012) identified putative cardiac niches in the heart epicardium and sub-epicardium with low oxygen tensions and housing a metabolically distinct population of glycolytic progenitor cells. Future attempts to characterize the stem cell niches in the heart atria and apex, however, are controversial. Nevertheless, cardiac niches may contain quiescent stem cells and progenitors in addition to the influence of all other heart cells (e.g., EC, CM, and SMC) that interacts and communicate together secreting a wide range of ECM proteins and soluble factors (reviewed by Aguilar-Sanchez et al., 2018).

Besides cell-to-cell interactions, ECM and soluble trophic factors present in the tissue microenvironment, other important modulators to consider are the biomechanical and electrical stimuli that influence the differentiation and maturation of cardiomyocytes, and the correct formation of the heart. Mechanical forces present during cardiac development include shear and strain stress, flow forces (i.e., blood flow), pressure and stretch. These mechanical signals are sensed by cells and converted into intracellular signals that activated pathways that induce gene expression and cellular commitment. The perception and transmission of signals involve both cell–cell and cell–ECM interactions (reviewed by Lindsey et al., 2014; Andrés-Delgado and Mercader, 2016; Stoppel et al., 2016). The mechanical force dynamics in the development of the heart involves, e.g., cFB that secrete a great amount of ECM proteins influencing the stiffness and topography of the organ while cardiomyocytes improves expression of sarcomeric proteins increasing the contractile stress and tissue strength. As a result, the heart pumps more blood, which increases the flow forces (reviewed by Majkut et al., 2014). All these biomechanical forces are interconnected and influence different processes during heart formation, from the proliferation and differentiation of cardiomyocytes to the correct formation of valves and cardiac chambers. Considering the importance of mechanical and electrical signals in cardiac development, many approaches have been developed in an attempt to understand and mimic these signals *in vitro*, with the aim of improving cardiomyocyte differentiation or maturation (reviewed by Zhu et al., 2014; Stoppel et al., 2016; Besser et al., 2018).

**FIGURE 3:**
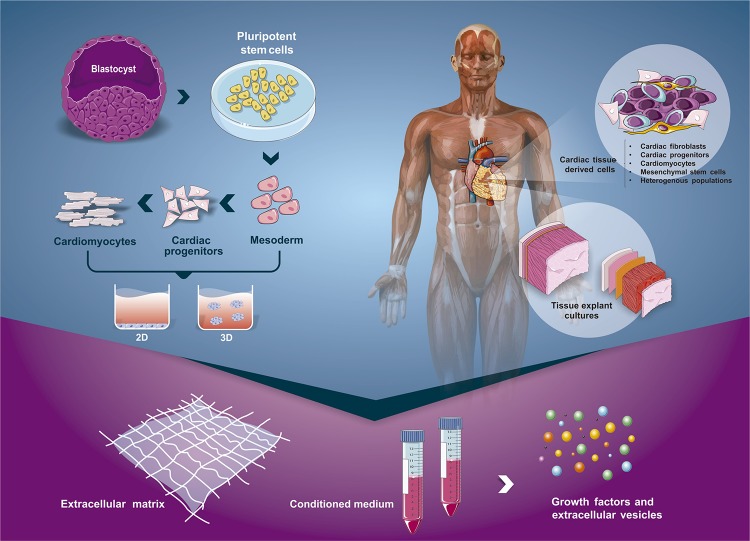
Scheme of different approaches to isolate the cardiac secretome. Extracellular matrix, soluble factors and extracellular vesicles can be obtained from the cardiomyogenic developmental process (using hPSC) or from adult tissues through the use of different strategies. The process of hPSC cardiac differentiation can occur using 2D (monolayer) or 3D (embryoid bodies) cultures. On the other hand, with fragments of neonate or adult heart, we could isolate specific cell populations from the tissue (e.g., fibroblasts, cardiac progenitors) or culture the entire or parts of the cardiac tissue.

Some possibilities to approach cell-niche signaling relies on the use of PSC cardiomyogenic modeling, culture of cardiac tissue explant/cardiospheres and isolated cell populations (e.g., cardiac progenitor cells). Figure 3 summarizes the current strategies used to model cardiomyogenesis or cardiac cell/tissue behavior *in vitro* and ways to analyze the cardiac secretome (CS). The secretome comprises the complex set of secreted molecules/vesicles from cells to the extracellular space. At the cell–environment interface, the biological activity of the secretome is exerted by the modulation of signal transduction pathways that direct fundamental biological processes on cells, including cell fate and proliferation (Reus et al., 2016). Through the analysis of the ECM composition, soluble factors and EVs derived from cell and tissue cultures, we can search for essential signals and understand regulatory networks underlying human heart development. Regarding this issue, genomic and proteomic studies have provided important contributions (Reus et al., 2016; Wolling et al., 2018; Leitolis et al., 2019; Pereira et al., 2019). In the next sections, we will describe approaches to study the composition of cardiac secretome and discuss strategies that use the extracellular signals to induce PSC cardiac differentiation and maturation of PSC-CM.

## Exploring the Cardiac ECM: Composition, Tissue Engineering Strategies and *in vitro* Modeling

Considering the actual challenges related to the use of PSC, the development of three-dimensional (3D) heart tissues constructs represents a strategy to improve *in vitro* models, both for studies concerning heart physiology and drug screening and to advance *in vivo* (translational) applications (Hirt et al., 2014; Ogle et al., 2016). However, recapitulating the complexity of cardiac tissue *in vitro* – structure, composition, and mechanical properties – is not an easy task. In this context, knowing the ECM, an important niche component, may have a relevant role in the development of new approaches to mimic heart tissue. Some methodologies use synthetic or natural biomaterials, singular ECM proteins or their combinations, and decellularized cardiac tissue (Smith et al., 2017; Zhang et al., 2017).

### Cardiac ECM Composition

The ECM is not an inert scaffold restricted to supporting and structuring cells in tissues or organs but is also an important modulator of cellular responses either through its direct interaction with cell receptors, through the control of growth factor activities, controlling their diffusion or release, and transmitting mechanical signals (Rozario and DeSimone, 2010). ECM proteins comprise approximately 1–1.5% of the mammalian proteome called the “core matrisome,” which is composed of approximately 300 proteins shared among glycoproteins, proteoglycans and many collagen types. In addition, there are a great number of ECM-associated proteins, including the group of mucins and lectins, enzymes, matrix metallopeptidases, and secreted factors, such as chemokines, interleukins, and growth factors (Hynes and Naba, 2012; Naba et al., 2012, 2016). Considering the diversity of ECM components, the content of ECM varies among tissues, phase of development and according to the pathophysiological state of an organ (Christalla et al., 2012; Bonnans et al., 2014; Kular et al., 2014).

The cardiac ECM composition and function have been studied over the last few years. Several collagen types, fibronectin, laminin, fibrillins, proteoglycans, such as perlecan, agrin and glypicans, and glycosaminoglycans, mainly hyaluronan, are among the constituents of heart ECM (Lockhart et al., 2011; Rienks et al., 2014). As a dynamic milieu, the expression of the heart ECM varies during cardiac development and, accordingly, development of the heart region. Furthermore, it was previously described that the deletion of some ECM proteins causes serious heart defects, many of them causing embryonic lethality, as reviewed by Lockhart et al. (2011). Another component present in the ECM, which is important in the development of the heart and in its response to injury, is matricellular proteins. Although these proteins do not have structural function, they interact with surface receptors, growth factors, and ECM, among others, aiming to integrate the signals of the microenvironment. Among the members of this group are thrombospondin, tenascin, SPARC (acid secreted protein and cysteine rich), osteopontin, periostin and members of the CCN family (Frangogiannis, 2012, 2017; Rienks et al., 2014; Rienks and Papageorgiou, 2016).

Among heart cells, cFB are the main producers of cardiac ECM proteins. They are responsible for the ECM homeostasis, maintaining the balance between production and degradation of ECM proteins that are essential for the correct heart function. Despite its importance, there is still a lack of knowledge about the surface markers that could reveal their real molecular identity and, even, about functionality during the development of the heart. The cFBs are mainly derived from cells of the epicardium (with some contribution of the endothelium) that undergo epithelial-to-mesenchymal transition. In addition to ECM proteins, they secrete soluble factors (as will be discussed later in this review) and are also important in transducing and responding to electromechanical signals (Furtado et al., 2016; Civitarese et al., 2017; Tallquist and Molkentin, 2017). Interestingly, it was described that cFB expressed many specific cardiogenic genes, different of fibroblasts from other sources (Furtado et al., 2016). In adult heart, several factors can affect cFB activation, including response to injury. In this case, cFB can differentiate into myofibroblasts, which have increased proliferative and secretory capacity. The raise in the number of cFB and/or the deposition of ECM proteins causes fibrosis and, consequently, changes the rigidity of the tissue, the interaction between the cells, impairs myocyte contractility, oxygenation and metabolism (Tirziu et al., 2010; Martin and Blaxall, 2012; Fountoulaki et al., 2015), completely changing the cardiac microenvironment.

Originally, to better understand the role of cardiac ECM in embryonic development and elucidate its composition, the majority of studies used murine and other animal models (chicken, zebrafish) or were based on data related to congenital human cardiac diseases (Lockhart et al., 2011). Recently, one alternative is the use of hPSC cardiomyogenic differentiation to investigate ECM proteins secreted by cells. High-throughput analysis during hPSC differentiation showed that genes related to the ECM are modulated throughout the cardiac commitment process (den Hartogh et al., 2016; Pereira et al., 2019). Additionally, it was verified that during CM differentiation, total proteoglycan and hyaluronan decreased (Chan et al., 2010); meanwhile, using an endogenous optical signal, it was showed that elastin increased until day 9 of the murine EB differentiation protocol, and type I collagen (Col I) increased over 12 days (Thimm et al., 2015). Using a spontaneous differentiation protocol and isolating the beating areas, it was demonstrated that hESC-CM were surrounded by types I, IV, and XVIII collagens, laminin isoforms and fibronectin (FN) (van Laake et al., 2010).

In an attempt to characterize the CPC niche, Schenke-Layland et al. (2011) isolated fetal mouse and human hearts and identified a population of CPC that expressed Isl1^+^Flk1^+^. This population was delineated by a basement membrane with, primarily, collagen type IV (Col IV) and laminin, while FN and Col I were also present but more distant from it. Considering these results, it was shown that murine ESC (mESC) monolayer cultures with Col IV and laminin increase the number of CPC (Flk1^+^), a number that was even higher in 3D Col IV cultures (Schenke-Layland et al., 2011).

### Myocardial Tissue Scaffolds

More recently, the understanding of the native ECM composition and its role in cell behavior has advanced due to decellularization approaches (Crapo et al., 2011). Decellularization techniques aimed to remove all cellular components of an organ or tissue, maintaining the ECM proteins and structure (Crapo et al., 2011; Tang-Quan et al., 2018). This approach leads to distinct possibilities, including the development of new cardiac tissue engineering strategies, through the use of decellularized/recellularized organs in transplants, as well as allowing the characterization of the cardiac ECM in normal or pathological conditions. Over the last 10 years, different decellularization and recellularization strategies were developed and performed with murine (Ott et al., 2008; Carvalho et al., 2012; Lu et al., 2013; Wang et al., 2019), porcine (Ott et al., 2008; Weymann et al., 2014; Ferng et al., 2017; Lee P.-F. et al., 2017), bovine (Arslan et al., 2018) and even human (Sánchez et al., 2015; Garreta et al., 2016; Guyette et al., 2016) heart tissue (revised by Scarrit et al., 2015; Tang-Quan et al., 2018).

Regarding ECM characterization, histochemical and immunofluorescent analyses were performed to visualize the ECM structure and some of its components. Large-scale proteomic analysis through mass spectrometry of matrix proteins could be difficult, since they are poorly soluble as a result of their macromolecular nature, extensive posttranslational modifications and the tendency to form protein complexes (Chang et al., 2016; Lindsey et al., 2018). Nevertheless, many approaches have sought to improve the protocols for mass spectrometry ECM characterization, allowing the identification of heart ECM proteins and collaborating with advances in the area (Lindsey et al., 2018). The number of extracellular proteins identified varies according to the form of ECM solubilization, analysis and equipment used.

Different extracellular protein combinations were identified after decellularization processes in the infarcted area of the mouse left ventricle (De Castro Brás et al., 2013), in normal porcine myocardium (Perea-Gil et al., 2018), after ischemia/reperfusion porcine heart injury (Barallobre-Barreiro et al., 2012), from rat hearts (Nguyen et al., 2018) and others. Human cardiac tissues were also characterized under normal conditions (Guyette et al., 2016; Johnson et al., 2016; Robert et al., 2017). For example, Johnson et al. (2016) used ECM-target and non-target methodologies to quantify 43 proteins in decellularized samples and identified more than 200 proteins in the global approach; these researchers also verified some variation in ECM composition between different heart donors. Despite the differences, in general, the matrisome components found in greater quantity in decellularized cardiac ECM are collagen types, glycoproteins, such as fibronectin, and members of the basal membrane, such as laminins and perlecan.

Rat neonatal CM, EC (rat or human origin), hCPC, mesenchymal stem cells (MSC), hPSC and hPSC-CM or hPSC-CPC were some of the cells used for tissue recellularization (Scarrit et al., 2015; Tang-Quan et al., 2018). Using undifferentiated hESC or hESC-derived mesendodermal cells, Ng et al. (2011) recellularized mouse hearts and, after 14 days under static culture conditions, cells began to express cardiac markers, such as cTnT, Nkx2.5, Myh6, and others. However, the cells were unable to contract, even *in vivo* (Ng et al., 2011). Lu et al. (2013) demonstrated that hiPSC-derived cardiac multipotent progenitors perfused into a decellularized mouse heart were able to migrate, proliferate and differentiate *in situ* into CM, SMC and EC, showing spontaneous contractions after 20 days, but they did not reach complete organ recellularization (Lu et al., 2013). Indeed, whole organ recellularization is an important barrier in cardiac tissue engineering. As a consequence, recent approaches include the use of cardiac patches with fragments/slices of decellularized matrices or its soluble form/hydrogel.

When undifferentiated mESC were cultured in slices of decellularized mouse hearts, they were able to express higher levels of cardiac markers in comparison with mESC cultured in slices of decellularized liver (Higuchi et al., 2013). Through the use of laser-cut sections of decellularized porcine myocardium, it was developed a system capable of maintaining hPSC-CM with organized sarcomeres and gap junctions that allowed the characterization of the biomechanical function of these EHT (Schwan et al., 2016). Pieces of decellularized rat heart were cultured with MSCs and hPSC-derived mature ventricular CM, which attached and formed a tissue-like structure with not only CM but also SMC and EC (Li et al., 2017). Other types of decellularized ECM were also tested both to support and differentiate PSC and to maintain PSC-CM. Hong et al. (2018) verified that decellularized mouse skeletal muscle was able to induce mESC to differentiate to a cardiac phenotype and supported mESC-CM (Hong et al., 2018). In addition, human placenta-derived hydrogel is another decellularized ECM that proved to be sufficient to maintain and generate more synchronized and electrically coupled hiPSC-CM (Francis et al., 2017).

Using human decellularized cardiac tissue, Oberwallner et al. (2015) showed that this ECM could support the mESC and iPSC and favored cardiac lineage differentiation (Oberwallner et al., 2015). More recently, slices of decellularized human heart cultured with PSC-CM presented spontaneous beating after 7–10 days, confirming cell–matrix interaction, demonstrated better electrophysiological response than in Matrigel and showed increased expression of cardiac ion channels in CM (Garreta et al., 2016). Additionally, Guyette et al. (2016), using two different tissue thicknesses and PSC-CM, showed spontaneous tissue contraction after 4–10 days, maintenance in culture for 60 or 120 days and the formation of a mechanical and electrical active myocardial tissue (Guyette et al., 2016).

Despite 3D approaches, an alternative is the use of hydrogels derived from decellularized ECM. Hydrogel prepared from decellularized porcine hearts combined with Col I, in proportion 75% ECM and 25% collagen, increased the number of cells expressing cTnT in comparison with those hydrogels with low ECM content (25%) or 100% collagen. This strategy also improved the maturation of hESC EBs, as demonstrated by the upregulation of connexin 43 (Cx43), the cardiac troponin I (cTnI) striation patterns, the improvement in the number of contracting cells and in the contraction amplitude (Duan et al., 2011). Fong et al. (2016) used hydrogels derived from decellularized bovine adult and fetal hearts in culture with hiPSC-CM in 2D (coating) and 3D (cardiac ECM + fibrinogen) approaches. These researchers demonstrated that 3D cultures with adult cardiac heart showed better improvement in CM maturation, mainly related to higher expression of mature cardiac genes (MYL2, Cx43, SERCA2a, and HCN4) and increased calcium signaling and kinetics compared with CM in 2D cultures (Fong et al., 2016). Together, these results indicate that native ECM provides a microenvironment capable of differentiating cells and providing a scaffold for the culture of CM, improving, at least in part, the maturation of these cells. These and other strategies used to induce differentiation and maturation of PSC-CM are summarized in Table 3.

**TABLE 3:** Summary of ECM approaches to improve PSC cardiomyogenic differentiation and/or PSC-CM maturation.

**Decellularized heart ECM**	**Method**	**Cells**	**Highlights**	**References**
Whole organ	Mouse ECM. Injected cells.	hESC or hESC-derived mesendodermal cells	After 14 days, both cell types expressed cardiac markers genes (cTnT, NKX2.5). No spontaneous contraction.	Ng et al., 2011
	Mouse ECM. Cells perfunded with growth factors.	hPSC-derived cardiac multipotent progenitors	Cells differentiate *in situ* in CM, SMC, and EC. Spontaneous contraction after 20 days. No complete recellularization.	Lu et al., 2013
	Human ECM. Injected cells. Human heart bioreactor.	hiPSC-CM	After 14 days, cells remained viable, integrate with matrix and showed a range of maturity. No complete recellularization.	Guyette et al., 2016
Slices	Mouse ECM. 60-μm thick slices.	mESC	Higher levels of cardiac markers in comparison with liver decellularized ECM.	Higuchi et al., 2013
	Porcine ECM. 150-μm thick slices. Laser-cut sheets.	hPSC-CM	Developed of an EHT for biomechanical characterization of PSC-CM. Cells presented organized sarcomeres and formed gap junctions.	Schwan et al., 2016
	Rat ECM.	MSCs and hPSC-derived ventricular CM	EHT with 75% CM and 25% MSC. After 2 weeks, cells formed a tissue-like structure with spontaneous contraction and CM, SMC and EC.	Li et al., 2017
	Human ECM from patients with end-stage non-ischemic dilated cardiopathy. 300-μm thick slices.	mESC, miPSC	Supported PSC proliferation. Increase expression of cardiac markers.	Oberwallner et al., 2015
	Human ECM. 400-μm thick slices.	PSC-CM	Spontaneous beating after 7–10 days. Better electrophysiological response. Uniform contraction, functional gap junctions. Increase expression of cardiac ion channels in CM.	Garreta et al., 2016
	Human ECM. 200-μm thick slices. Cardiac fiber bundles with 15 mm length, 2.5 mm diameter. Injected cells.	PSC-CM	Cells adhered, remained viable and functional. Spontaneous beating after 4–10 days. Maintenance in culture for 60–120 days. Formation of mechanical and electrical tissue.	Guyette et al., 2016
Hydrogel	Different hydrogels composition: 75%, 25% or 0% of porcine ECM.	hESC EBs	The hydrogel composed of 75% porcine ECM:25% collagen increase the number of cells cTnT + Improve expression of Cx43, number of contracting cells and contraction amplitude.	Duan et al., 2011
	2D (coating) or adult (cardiac patch) bovine adult and fetal ECM	hiPSC-CM	3D cultures with adult tissue showed higher expression of mature cardiac genes. Increase calcium signaling.	Fong et al., 2016
3D bioprinting	Porcine ECM. Different bioinks composition.	hCPC and/or MSC	Improve maturation of CPC. MSC + VEGF promoted vascular formation.	Jang et al., 2017
	Decellularized porcine ECM bioink. Custom digital light processing (DLP)-based scanningless and continuous 3D bioprinter.	hiPSC-CM	Improve expression of mature cardiac genes.	Yu et al., 2019
**ECM preparations**				
ECM proteins	Different proportions of fibronectin and laminin.	hESC	Ratio Fibronectin and Laminin (70:30) improve the number of differentiated cells (higher than 60% cTnI +).	Sa et al., 2014
	Systematic optimization of different ratios of type I collagen, laminin and fibronectin.	miPSC	Hydrogel composed of 61% type I collagen, 24% laminin-111 and 15% fibronectin increase number of cells (cTnT+).	Jung et al., 2015
	Different combinations of laminin, fibronectin, types I, III and IV collagens.	hESC- derived CPC	Combinations of ECM proteins improve CPC attachment and survival. Fibronectin, types I, III and IV collagens showed better results.	Lu et al., 2017
	Development of a Laminin-221 based cardiac differentiation protocol.	hESC	Combination of LN-521 + 221 matrix generated more CM (∼80%). High reproducibility confirmed by bulk and single-cell RNA-seq.	Yap et al., 2019
	Biowire platform: Cells culture in type I collagen gel around a suture in a PDMS channel. Associate with electrical stimulation.	hPSC-CM and non-CM cells (e.g., FB, SMC, EC).	CM increase size, rod-like shape, organized sarcomeric banding, lower proliferative rate, improved Ca(+2) handling.	Nunes et al., 2013
Matrigel	Cells encapsulated in 3D cardiac strips composed of matrigel and type I collagen. Associated with mechanical cyclic stretch.	hESC-CM associated or not with non-CM cells	Conditions with non-CM cells improve more CM maturation. Cyclic stretch improved sarcomere size and expression of mature cardiac genes.	Zhang et al., 2017
	Cardiopatch hydrogel composed of matrigel, fibrinogen and cardiac media.	hPSC-CM	Improve expression of mature cardiac genes, sarcomeric banding, lower proliferative rate, more mature electrophysiology.	Shadrin et al., 2017
	Coating with fibronectin or matrigel in glass coverslips or PDMS membranes.	hPSC-CM	Matrigel in PDMS improve CM electrophysiology, number of binucleated cells, expression of sarcomere and myofilament markers.	Herron et al., 2016
	Matrigel Matress: 0.4–0.8 mm-thick of undiluted matrigel.	hiPSC-CM	CM developed rod-shape morphology, increase sarcomere size, upstroke velocity and expression of cardiac markers.	Feaster et al., 2015
	Cells culture in matrigel or hyaluronan-based hydrogel associated or not with pro-survival factors.	hiPSC-CM	*In vivo*, CM in matrigel presented a more mature phenotype.	Ogasawara et al., 2017
Fibrin	3D fibrin cardiac patch.	hESC-CM (SIRPA cells)	Improve sarcomere size, conduction velocity and expression of cardiac genes.	Zhang et al., 2013
	3D fibrin matrix.	hiPSC-CM	Increase sodium current density and upstroke velocity.	Lemoine et al., 2017
	Fibrin hydrogels associated with stretch and electrical stimulation	hiPSC-CM	Early-stage iPSC-CM associated with physical conditioning at an increasing intensity accelerated maturation, showed superior electrophysiological properties, CMs with increase cell size and sarcomere length.	Ronaldson-Bouchard et al., 2018

*EBs, embryoid bodies; CM, cardiomyocytes; SMC, smooth muscle cells; EC, endothelial cells; VEGF, vascular endothelial growth factor; MSC, mesenchymal stem cells; CPC, cardiac progenitor cells.*

### Isolated ECM Components

In addition to the use of complex matrices and based on previous knowledge regarding cardiac ECM components, studies were performed with isolated ECM components. Many studies have used different combinations of ECM proteins to improve cardiac differentiation. hESC differentiated in a specific ratio of fibronectin (FN) and laminin (70:30) showed more differentiated cells than gelatin cultures, and its effects may be related to the integrin-mediated MEK/ERK signaling pathway (Sa et al., 2014). Furthermore, to optimize miPSC cardiac differentiation, Jung et al. (2015) investigated various combinations of ECM proteins. A systematic optimization indicated that a solution containing 61% Col I, 24% laminin-111 and 15% FN increased the number of cells expressing cTnT, MHCa, and α-actinin compared with suboptimal solutions (Jung et al., 2015). Additionally, it was demonstrated that the combination of ECM components, including FN, types I, III, and IV collagens, was important to allow hESC-derived cardiac progenitors to attach and survive (Lu et al., 2017). Recently, Yap et al. (2019), after verifying the higher expression of laminin-221 (LN-221) in adult cardiac tissue, developed a LN-221-based cardiac differentiation protocol, which reached more than 80% TNNT2^+^ cells and presented high reproducibility with 2 different hESC lines. Additionally, the cardiac progenitors generated during this process were able to improve cardiac function in mice after myocardial infarction (Yap et al., 2019).

In addition to affecting PSC cardiac differentiation, niche components and structure could also be used to stimulate the maturation of PSC-CM. Then, studies have attempted to use both 2D or 3D cultures with synthetic or natural matrices to reach this goal. To generate a microenvironment favorable to CM maturation, hPSC-CM and non-CM cells (as FB, SMC, and EC) were seeded in a Col I gel localized around a template suture in a poly(dimethylsiloxane) (PDMS) channel – a platform called “biowire.” Using this 3D structure associated with electrical stimulation, CMs increased their size, acquired a characteristic rod-like shape and an organized sarcomeric banding, presented a lower proliferative rate and improved Ca^+2^ handling properties. This finding indicates that the “biowire” platform with electrical stimulus (6 Hz) was able to induce hPSC-CM to a more mature phenotype (Nunes et al., 2013).

Comparisons of 2D and 3D cultures were performed by other groups. Using a 3D fibrin-based cardiac patch, differentiated EBs were dissociated and seeded with different percentages of SIRPA^+^ cells (CM marker) on the patch. Compared to monolayer cultures, 3D scaffolds augmented the conduction velocity, the size of sarcomeres and the expression of cardiac genes in hESC-CM (Zhang et al., 2013). Also, 3D cardiac strips produced through encapsulation of hESC-CM – with and without other niche cells (FB or MSC) – in Matrigel and Col I, associated with mechanical cyclic stretch, demonstrated potential in mature CM. Although all the conditions could generate a level of maturation in CM, those cultured with MSC or FB presented a more mature phenotype. Additionally, the use of cyclic stretching improved the sarcomere length and gene expression of maturation markers (Zhang et al., 2017).

Another platform of 3D construct is the “cardiopatch,” a hydrogel composed of fibrinogen, Matrigel and cardiac media mixed with CM. During 5 weeks of differentiation, hPSC-CM presented sarcomeric banding (including M-band and T-tubules), enhanced expression of markers related to the cardiac maturation process (e.g., TNNI3, MYL2, CASQ2, and CKM), progressive decrease in cell proliferation, higher conduction velocity, among others (Shadrin et al., 2017). Comparing a 3D EHT generated with hiPSC-CM in a fibrin matrix with 2D monolayer cultures, Lemoine et al. (2017) demonstrated that the EHT strategy increased sodium current density and upstroke velocity, both values more similar to those from adult human CM (Lemoine et al., 2017). Furthermore, early-stage hiPSC-CM incorporated into fibrin hydrogels subjected to stretch and electrical stimulation (increase intensity training) accelerated CM maturation, confirmed by expression of mature cardiac makers, increase cell size and sarcomere length and mature-like electrophysiological responses (Ronaldson-Bouchard et al., 2018).

The Matrigel^®^, a commercially available protein mixture used in maintenance of PSC and cardiac differentiation protocol (Zhang J. et al., 2012), was also applied to different strategies, alone or in combination with specific ECM proteins. For example, Herron et al. (2016) tested different ECM combinations to improve the maturation of hPSC-CM: coating of FN or Matrigel in glass coverslips or PDMS membranes. Matrigel + PDMS promoted greater CM maturation. Parameters such as conduction and upstroke velocities, electrophysiological, number of binucleated and proliferated cells, expression of mature sarcolemal and myofilament markers resemble that of mature CM under optimal conditions (Herron et al., 2016). Another strategy developed was the “Matrigel Mattress.” In this method, 0.4- to 0.8-mm-thick undiluted Matrigel^®^ was prepared, and the hiPSC-CM cultured on this substrate developed a more rod-shaped morphology, increased sarcomere length, upstroke velocity and expression of cardiac markers (Feaster et al., 2015).

This type of methodology could be combined with *in vivo* maturation. For example, hiPSC-CM was diluted on Matrigel with pro-survival factors or on a hyaluronan-based hydrogel associated or not with pro-survival factors and transplanted into an infarcted rat. After 4 weeks, the CM cultured on Matrigel + factors presented a more mature phenotype than the other groups (Ogasawara et al., 2017). Kadota et al. (2017) compared cells transplanted in neonatal rat hearts uninjured and infarcted adult rat hearts. In all situations, the hiPSC-CM engrafted and survived in rat hearts, but it was the adult tissue that promoted faster CM maturation.

Among the most recent strategies to produce an environment for CM maturation is the use of 3D printing technologies. For instance, 3D printing allows the building of specific patterns of 3D constructs (Ma et al., 2018) and the use of solubilized decellularized ECM as bioink to culture with CPC (Jang et al., 2017) or iPSC-CM (Noor et al., 2019; Yu et al., 2019) to improve maturation of the cells. Considering the results discussed in this study, we showed that mimic ECM microenvironmental signals using 3D cultures, combinations of ECM proteins or a natural scaffold are important modulators that influence cardiac differentiation or maturation.

## Cardiac Soluble Factors: Effects on Cardiomyocyte Fate and Behavior

To maintain their functions properly, chemical signaling in the heart should be orchestrated by a variety of signals released by myocyte and non-myocyte cells. As mentioned previously, the set of those signals constituted by secreted soluble factors comprises the CS. CS include several bioactive molecules, such as growth factors, endocrine hormones, cytokines and peptides (Doroudgar and Glembotski, 2012). In addition, CS may also contain extracellular vesicles (EVs) that load different types of cargo, which may vary with biogenesis, cell type, and physiological conditions (Abels and Breakefield, 2016). Because of the complex interplay between cardiac factors, cell communication in the heart has not been fully elucidated to date. However, extensive evidence indicates that the secretome from cardiac cells may influence CM development and behavior.

One approach to studying and decipher the actors of cellular communication in the heart is the analysis of the conditioned medium from *in vitro* cultures of cardiac cells (Figure 3), such as cardiospheres (Sharma et al., 2015), cardiac resident cells (Zhang et al., 2015; Reus et al., 2016) and heart explant tissues (Schittini et al., 2010). In the last several years, mass spectrometry approaches have been used for signaling investigation. According to Lindoso et al. (2016), this approach provides an overview of proteins present in media and enables a measurement of protein level changes during normal or pathophysiological conditions. Recently, a study analyzed the secretome collected at seven different time points during *in vitro* CM differentiation. The authors found 1802 proteins significantly regulated during differentiation of which 431 are annotated as secreted. Numerous proteins that remarkably vary during the differentiation process affect the Wnt, TGFβ, Activin A, Nodal, BMP and FGF signaling pathways (Wolling et al., 2018). The identification of paracrine factors that modulate CM differentiation, proliferation and maturation may help the development or improvement of protocols for the *in vitro* differentiation of CM from PSC. In this section, we provide a broad overview of recent investigations of paracrine factors that affect CM with an emphasis on those released by the main types of cardiac cells. We also explore studies involving EVs and its influence in cardiac cell behavior.

### Signaling From Cardiac Resident Cells

#### Endothelial Cells

EC constitute an important component of the heart. Recently, ECs were identified as one of the most abundant cell types in this organ (Pinto et al., 2016). Anatomically, these cells form a monolayer that covers the heart cavities and compose the vascular network that perfuses the myocardium. Therefore, factors secreted by ECs, such as NO, endothelin-1, angiotensin II (Ang II), prostaglandins, natriuretic peptides, adenyl purines, neuregulin-1, FGF, and VEGF, are able to affect heart function (Shah, 1996; Tirziu et al., 2010). Recently, the mediators of the EC-CM interaction involved in cardiac remodeling and regeneration were summarized in a review (Talman and Kivelä, 2018). In fact, the communication between ECs and CM is crucial for the maintenance of cardiac homeostasis, and the disruption in this signalization can result in pathophysiological conditions (Gogiraju et al., 2019). Furthermore, CM generation was demonstrated to be affected by the niche provided by ECs (Chen et al., 2010), and the ability of ECs to enhance the maturity of hPSC-CM was already verified (Caspi et al., 2007; Tulloch et al., 2011; Ravenscroft et al., 2016). Kivelä et al. (2019) also demonstrated that ECs regulate physiological CM growth via VEGFR2. Recently, a study generated a human cardiac microtissue through the co-differentiation of CM and ECs from PSC (Giacomelli et al., 2017). Interestingly, the inclusion of ECs (generated with a cardiac identity) and prolonged time in culture induced changes in CM gene expression associated with CM maturation. Among the EC-CM mediators, neuregulin-1 (NRG-1) is one of the key players in CM development (Rupert and Coulombe, 2015). Previous studies reported that NRG-1β/ErbB signaling was able to increase cardiomyogenesis in mESC and participate in cardiac subtype (“working-type” atrial or nodal) selection (Chen et al., 2013). Beyond the individual effects of NRG-1, this molecule also acts synergistically with IGF-1 to enhance proliferation and metabolic maturity in CM (Rupert and Coulombe, 2017). Other soluble factors released by ECs are able to drive cardiac differentiation. Endothelin-1 added to Nkx2.5 + CPC culture induced CPC differentiation into cardiac pacemaking cells (Zhang X. et al., 2012). This molecule has also been suggested to stimulate terminal differentiation of CM (Paradis et al., 2014). In addition, brain natriuretic peptide (BNP), a cardiac hormone secreted by EC, CM and cFB, stimulated CPC proliferation and CM differentiation, and these effects were demonstrated to occur via BNP binding to NPR-A and NPR-B, respectively (Bielmann et al., 2015).

#### Cardiac Fibroblasts

The cFBs are cells that produce connective tissues and are recognized as modulators of cardiac function, development and homeostasis (Zhang P. et al., 2012; Ivey and Tallquist, 2017). As mentioned previously, cFB are responsible for the synthesis of the major part of ECM proteins in the heart. Therefore, the main interplay between cFB-CM communication appears to occur through ECM molecules secreted by cFBs. These cells also secrete proteins related to cardiac development, such as FGFs, TGF, Ang II, interleukin-6 (IL-6), and IL-33 (Kakkar and Lee, 2010; Takeda and Manabe, 2011). In fact, conditioned medium from ventricular cFBs was demonstrated to induce Nkx2.5^+^ CPC differentiation in CM through Wnt pathway activation (Zhang et al., 2015). Similarly, our group investigated the effects of conditioned medium from CRSCs (a population that includes DDR-2^+^ cells) on progenitor cell (H9c2) behavior. The CRSC secretome was able to drive the proliferation and cardiac differentiation of H9c2 cells (Reus et al., 2016). Furthermore, the cFB conditioned medium was also reported to influence CM phenotype, including hypertrophy, expression of vimentin and electrophysiological changes (LaFramboise et al., 2007; Pedrotty et al., 2009). Recently, the secretome of cFB was investigated in normal and stressed (hypoxic) conditions (Cosme et al., 2017). The conditioned media was separated to obtain exosome and exosome-depleted fractions, and the results revealed almost 494 proteins differentially expressed between fractions and oxygen conditions. Indeed, culture conditions interfere with cFB secretion and consequently in the CM response. Furthermore, cFB can be activated in response to tissue injury and present a phenotype characterized by the expression of alpha-smooth muscle actin (α-SMA) (Hu and Phan, 2013). Cartledge et al. (2015) demonstrated hypertrophic effects on CM cocultivated with cFB, myofibroblast and myofibroblast-conditioned medium, which were related to TGF-β released from cFB. In fact, CM hypertrophy induced by cFB-paracrine factors has been extensively described (Booz et al., 1999; LaFramboise et al., 2007). In addition, in damaged myocardium, activated cFB also releases proinflammatory cytokines, such as cardiotrophin-1 (CT-1), a member of the IL-6 family. This molecule could enhance mouse IPS cardiomyogenic differentiation partly via the JAK2/STAT3/Pim-1 pathway and stimulate CM maturation (Liu et al., 2015).

#### Cardiac Progenitor Cells

Cardiac progenitor cells are a heterogeneous group of cells distributed throughout the heart. These cells are extensively studied mainly because of their potential effects on injured cardiac tissue (reviewed by Le and Chong, 2017). Originally, it was believed that after transplantation, CPC would be able to restore cardiac function through differentiation in CM, EC, and SMC (Torella et al., 2006; Bearzi et al., 2007). However, recent reports suggest that the regenerative effects induced by CPC occur through secreted paracrine factors (Khanabdali et al., 2016; Le and Chong, 2017). Many subtypes of cardiac stem cells have been reported (Chong et al., 2014), and apparently, the paracrine factors released are also diversified between them; however, the therapeutic effects occur with the different CPC populations. Recently, Torán et al. (2019) aimed to define a set of proteins specifically secreted by CPC. Authors isolated human CPC from myocardial samples and conducted a proteomic assay. The analysis identified a group of factors expressed at high to medium levels by CPC that included IL-1, GROa (CXCL1), CXCL6 (GCP2), and IL-8 (Torán et al., 2019). Similarly, considering the effects of CPC in myocardial recovery, Sharma et al. (2017) isolated cardiac progenitor cells from neonatal (nCPC) and adult patients (aCPC) and compared the functionality of their conditioned medium. After extensive characterization of CPC paracrine factors, differences were found in the secretion profile during development that affected the ability of conditioned medium to recover myocardial function. Both secretomes significantly induced proliferation and reduced apoptosis in CM; however, nCPC conditioned medium was more effective than aCPC (Sharma et al., 2017). A recent study also profiled the proteins that were secreted by CPC (Sca−1^+^) derived from healthy and transgenic heart failure mice aiming to define the factors that are modulated in a failing heart microenvironment. The results showed that proteins usually associated with tissue regeneration, such as CSF1, COCA, IBP6, and TCPG, were found to be more abundant in transgenic samples than in healthy samples (Samal et al., 2018).

### Extracellular Vesicles Signaling: Effects on Cardiomyocytes and Cardiac Tissue

In the heart, as well as in other tissues, protein secretion occurs in different ways: (1) direct release in the extracellular space through membrane-derived secretory vesicles; (2) translocation of cytosolic proteins across the plasma membrane; and (3) packaging in EVs (Reviewed by Doroudgar and Glembotski, 2012). The last mechanism is becoming more studied over the last several years. Since the discovery that EVs may harbor a varied content that includes not only proteins but also other active molecules (miRNAs, mRNA, DNA, and lipids) (Abels and Breakefield, 2016), the understanding of cell signaling was brought to a higher level of complexity. EVs have been isolated from different sources, including the previously mentioned cells: EC (Piryani et al., 2019), cFB (Borosch et al., 2017) and CPC (Barile et al., 2014), as well as other important components of the cardiac microenvironment, such as ECM (An et al., 2017), MSCs (Angulski et al., 2017), and CM (Liu et al., 2018). Recently, we isolated and characterized EVs from different regions of human heart tissue. Our results demonstrated that cardiac EVs contain a set of proteins advantageous for tissue regeneration approaches, and we verified their potential to modulate proliferation, wound healing, adhesion and angiogenesis differently depending on the target cell type (Leitolis et al., 2019). A systematic review compiled studies that investigated the cardioprotective characteristics of EVs (Wendt et al., 2018). In fact, EVs are able to regulate many biological activities, including those related to CM. Cardiac progenitor cell-derived EVs have been shown to stimulate migration and proliferation in AC16 CM (Hocine et al., 2019) and inhibit CM apoptosis (Barile et al., 2014) through a mechanism that involves PAPP-A (pregnancy-associated plasma protein-A) (Barile et al., 2018). Similarly, Liu et al. (2018) showed that CM-derived EVs not only decreased CM apoptosis 24 h after rat infarction but also reduced arrhythmia and hypertrophy postinfarction (Liu et al., 2018). The hypertrophic effect on CM was also verified through cFB-derived exosomes in a paracrine mechanism by which Ang II intensifies its own signaling in CM (Lyu et al., 2013). In addition, apoptotic bodies (AB) also appear to modulate myocyte activity. CM-derived AB were able to stimulate proliferation and differentiation of CM precursors, as well as their frequency of contraction (Tyukavin et al., 2015). Beyond the studies conducted with EVs derived from the sources mentioned above, many studies have shown the biological effects of MSC-derived EVs (MSC-EVs). To date, many reports have demonstrated that MSC-EVs are able to exert protective effects in cardiovascular diseases, especially by the delivery of miRNA content to recipient cells. Recently, Moghaddam et al. (2019) summarized the cardioprotective exosomal miRNAs that include those secreted by MSCs. For instance, Wang et al. (2018) demonstrated that cardiac stem cells treated with MSC-derived exosomes containing miR-214 showed decreased apoptosis and reactive oxygen species (ROS) production after oxidative stress injury (Wang et al., 2018). Similarly, a recent study showed that exosomal miR-21-5p increased cardiac calcium handling and thereby contractility via the PI3K signaling cascade in human engineered cardiac tissue (Mayourian et al., 2018). Furthermore, a novel study showed that the coculture of iPSC-CM with MSCs modulates the functionality and maturation of CMs. These effects can be partially explained by the miRNA content in MSC-derived EVs (Yoshida et al., 2018). In fact, CM differentiation and maturation are closely related to miRNA regulation (White et al., 2016; Lock et al., 2018). For instance, let-7 family miRNAs were found to be highly upregulated during CM maturation, and let-7 members control CM metabolism, cell size and force contractility (Kuppusamy et al., 2015).

## Perspectives

The elements from microenvironmental signaling, such as ECM proteins and paracrine factors, both secreted by cardiac resident cells, are biologically active molecules capable of affecting CM commitment, subtype specification, proliferation and maturation. These molecules are being tested in coculture experiments (CM plus non-myocyte cells), as well as in assays that use total secretome (conditioned medium), EVs, decellularized heart ECM or combinations of isolated ECM proteins. In most cases, the molecular mechanisms by which these molecules act have not been fully decoded.

Despite the progress in understanding the cardiac niche in different development stages, the modulation of extracellular signaling and how it governs cardiac commitment has not been thoroughly elucidated to date. The factors mentioned in this review, as well as others not explored to date, may be important tools for modulation of *in vitro* cardiomyogenesis, mainly regarding maturation of the developed CM. Hence, improving the functional characteristics of CM could potentiate their use in regenerative medicine and facilitate further advances in cell therapy and tissue engineering.

## Author Contributions

All authors listed have made a substantial, direct and intellectual contribution to the work, and approved it for publication.

## Conflict of Interest Statement

The authors declare that the research was conducted in the absence of any commercial or financial relationships that could be construed as a potential conflict of interest.
